# Effectiveness of COVID-19 Pfizer-BioNTech BNT162b2 mRNA Vaccination in Preventing COVID-19–Associated Emergency Department and Urgent Care Encounters and Hospitalizations Among Nonimmunocompromised Children and Adolescents Aged 5–17 Years — VISION Network, 10 States, April 2021–January 2022

**DOI:** 10.15585/mmwr.mm7109e3

**Published:** 2022-03-04

**Authors:** Nicola P. Klein, Melissa S. Stockwell, Maria Demarco, Manjusha Gaglani, Anupam B. Kharbanda, Stephanie A. Irving, Suchitra Rao, Shaun J. Grannis, Kristin Dascomb, Kempapura Murthy, Elizabeth A. Rowley, Alexandra F. Dalton, Malini B. DeSilva, Brian E. Dixon, Karthik Natarajan, Edward Stenehjem, Allison L. Naleway, Ned Lewis, Toan C. Ong, Palak Patel, Deepika Konatham, Peter J. Embi, Sarah E. Reese, Jungmi Han, Nancy Grisel, Kristin Goddard, Michelle A. Barron, Monica Dickerson, I-Chia Liao, William F. Fadel, Duck-Hye Yang, Julie Arndorfer, Bruce Fireman, Eric P. Griggs, Nimish R. Valvi, Carly Hallowell, Ousseny Zerbo, Sue Reynolds, Jill Ferdinands, Mehiret H. Wondimu, Jeremiah Williams, Catherine H. Bozio, Ruth Link-Gelles, Eduardo Azziz-Baumgartner, Stephanie J. Schrag, Mark G. Thompson, Jennifer R. Verani

**Affiliations:** ^1^Kaiser Permanente Vaccine Study Center, Kaiser Permanente Northern California Division of Research, Oakland, California; ^2^Division of Child and Adolescent Health, Department of Pediatrics, Columbia University Vagelos College of Physicians and Surgeons, New York, New York; ^3^Department of Population and Family Health, Columbia University Mailman School of Public Health, New York, New York; ^4^NewYork-Presbyterian Hospital, New York, New York; ^5^Westat, Rockville, Maryland; ^6^Baylor Scott & White Health, Temple, Texas; ^7^Texas A&M University College of Medicine, Temple, Texas; ^8^Children's Minnesota, Minneapolis, Minnesota; ^9^Center for Health Research, Kaiser Permanente Northwest, Portland, Oregon; ^10^Department of Pediatrics, University of Colorado Anschutz Medical Campus, Aurora, Colorado; ^11^Center for Biomedical Informatics, Regenstrief Institute, Indianapolis, Indiana; ^12^Indiana University School of Medicine, Indianapolis, Indiana; ^13^Division of Infectious Diseases and Clinical Epidemiology, Intermountain Healthcare, Salt Lake City, Utah; ^14^CDC COVID-19 Emergency Response Team; ^15^HealthPartners Institute, Minneapolis, Minnesota; ^16^Fairbanks School of Public Health, Indiana University, Indianapolis, Indiana; ^17^Department of Biomedical Informatics, Columbia University Irving Medical Center, New York, New York;^18^Regenstrief Institute, Indianapolis, Indiana; ^19^Vanderbilt University Medical Center, Nashville, Tennessee.

The efficacy of the BNT162b2 (Pfizer-BioNTech) vaccine against laboratory-confirmed COVID-19 exceeded 90% in clinical trials that included children and adolescents aged 5–11, 12–15, and 16–17 years (*1*–*3*). Limited real-world data on 2-dose mRNA vaccine effectiveness (VE) in persons aged 12–17 years (referred to as adolescents in this report) have also indicated high levels of protection against SARS-CoV-2 (the virus that causes COVID-19) infection and COVID-19–associated hospitalization (*4*–*6*); however, data on VE against the SARS-CoV-2 B.1.1.529 (Omicron) variant and duration of protection are limited. Pfizer-BioNTech VE data are not available for children aged 5–11 years. In partnership with CDC, the VISION Network* examined 39,217 emergency department (ED) and urgent care (UC) encounters and 1,699 hospitalizations^†^ among persons aged 5–17 years with COVID-19–like illness across 10 states during April 9, 2021–January 29, 2022,^§^ to estimate VE using a case-control test-negative design. Among children aged 5–11 years, VE against laboratory-confirmed COVID-19–associated ED and UC encounters 14–67 days after dose 2 (the longest interval after dose 2 in this age group) was 46%. Among adolescents aged 12–15 and 16–17 years, VE 14–149 days after dose 2 was 83% and 76%, respectively; VE ≥150 days after dose 2 was 38% and 46%, respectively. Among adolescents aged 16–17 years, VE increased to 86% ≥7 days after dose 3 (booster dose). VE against COVID-19–associated ED and UC encounters was substantially lower during the Omicron predominant period than the B.1.617.2 (Delta) predominant period among adolescents aged 12–17 years, with no significant protection ≥150 days after dose 2 during Omicron predominance. However, in adolescents aged 16–17 years, VE during the Omicron predominant period increased to 81% ≥7 days after a third booster dose. During the full study period, including pre-Delta, Delta, and Omicron predominant periods, VE against laboratory-confirmed COVID-19–associated hospitalization among children aged 5–11 years was 74% 14–67 days after dose 2, with wide CIs that included zero. Among adolescents aged 12–15 and 16–17 years, VE 14–149 days after dose 2 was 92% and 94%, respectively; VE ≥150 days after dose 2 was 73% and 88%, respectively. All eligible children and adolescents should remain up to date with recommended COVID-19 vaccinations, including a booster dose for those aged 12–17 years.

VISION Network VE methods have been previously published (*7*). In brief, eligible medical encounters were defined as ED and UC encounters and hospitalizations among persons aged ≥5 years with a COVID-19–like illness diagnosis^¶^ who had received SARS-CoV-2 molecular testing (primarily by reverse transcription–polymerase chain reaction assay) during the 14 days before through 72 hours after the encounter. For adolescents aged 16–17 years, the study period began when COVID-19 vaccines were recommended and became available to persons aged ≥16 years at each study site (April–May 2021).** For children aged 5–11 years and adolescents aged 12–15 years, the study period began 5 weeks after the Pfizer-BioNTech vaccine was recommended for their age group.^††^ The dates when the Delta and Omicron variants became predominant (accounted for >50% of sequenced viruses) were determined for each study site based on state and national surveillance data.^§§^ Patients were excluded if they 1) were vaccinated before the CDC recommendation date for their age group, 2) received a third dose before booster doses were recommended for their age group, 3) received a booster dose <5 months after dose 2, 4) received 1 or >3 doses of the vaccine, or 5) if <14 days had elapsed since receipt of dose 2 or <7 days since dose 3. Patients who were likely immunocompromised based on diagnosis codes were also excluded.^¶¶^ VE was estimated using a case-control test-negative design comparing the odds of a positive SARS-CoV-2 test result between vaccinated (received 2 doses ≥14 days earlier or 3 doses ≥7 days earlier) and unvaccinated (received no doses) patients using multivariable logistic regression models*** (*7*). VE was not calculated for exposure categories with fewer than 20 encounters or with no SARS-CoV-2 test–positive cases. A statistically significant difference in VE or distributions of vaccination or infection status was indicated by nonoverlapping 95% CIs or standardized mean or proportion differences ≥0.2. All statistical analyses were conducted using R software (version 4.1.2; R Foundation). This study was reviewed and approved by the institutional review boards at participating sites or under a reliance agreement with the Westat, Inc. institutional review board.^†††^

## Emergency Department and Urgent Care Encounters

Among 39,217 eligible encounters at 306 ED and UC facilities, 23.4%, 46.2%, and 30.3% were among persons aged 5–11, 12–15, and 16–17 years, respectively (Table 1). Most encounters among adolescents aged 12–15 years and 16–17 years occurred during the Delta predominant period (14,491 [79.9%] and 8,800 [74.0%], respectively); among children aged 5–11 years, most (6,424 [70.0%]) occurred during the Omicron predominant period, reflecting differences in the dates when vaccines became available for the respective age groups.

**TABLE 1:** Characteristics of emergency department and urgent care encounters among children aged 5–17 years with COVID-19–like illness,* by COVID-19 Pfizer-BioNTech vaccination status^†^ and SARS-CoV-2 test result — 10 states,^§^ April 2021–January 2022

Characteristic	Total no. (column %)	No. (row %)				SMD^¶^	No. (row %)	SMD^¶^
		Pfizer-BioNTech vaccination status					Positive SARS-CoV-2 test result	
		Unvaccinated	2 doses (14–149 days earlier)	2 doses (≥150 days earlier)	3 doses (≥7 days earlier)			
**All ED and UC encounters**	**39,217**	**28,084 (71.6)**	**7,821 (19.9)**	**3,238 (8.3)**	**74 (0.2)**	**—**	**9,252 (23.6)**	**—**
Variant predominance period**								
Pre-Delta	955 (2.4)	851 (89.1)	104 (10.9)	0 (—)	0 (—)	0.84	113 (11.8)	0.81
B.1.617.2 (Delta)	26,048 (66.4)	17,596 (67.6)	6,496 (24.9)	1,954 (7.5)	2 (0.0)		3,655 (14.0)	
B.1.1.529 (Omicron)	12,214 (31.1)	9,637 (78.9)	1,221 (10.0)	1,284 (10.5)	72 (0.6)		5,484 (44.9)	
**Site**								
Baylor Scott & White Health	4,408 (11.2)	3,932 (89.2)	313 (7.1)	163 (3.7)	0 (—)	0.83	1,653 (37.5)	0.48
Columbia University	1,564 (3.9)	1,260 (80.6)	226 (14.5)	77 (4.9)	1 (0.1)		510 (32.6)	
HealthPartners	2,089 (5.3)	988 (47.3)	844 (40.4)	257 (12.3)	0 (—)		231 (11.1)	
Intermountain Healthcare	12,993 (33.1)	8,314 (64.0)	3,274 (25.2)	1,372 (10.6)	33 (0.3)		2,002 (15.4)	
Kaiser Permanente Northern California	2,287 (5.8)	1,134 (49.6)	795 (34.8)	339 (14.8)	19 (0.8)		578 (25.3)	
Kaiser Permanente Northwest	1,508 (3.8)	841 (55.8)	447 (29.6)	212 (14.1)	8 (0.5)		354 (23.5)	
Regenstrief Institute	7,374 (18.8)	6,008 (81.5)	972 (13.2)	384 (5.2)	10 (0.1)		2,391 (32.4)	
University of Colorado	6,994 (17.8)	5,607 (80.2)	950 (13.6)	434 (6.2)	3 (0.0)		1,533 (21.9)	
**Age group, yrs**								
5–11	9,181 (23.4)	8,599 (93.7)	582 (6.3)	0 (—)	0 (—)	1.07	2,776 (30.2)	0.20
12–15	18,138 (46.2)	12,064 (66.5)	4,547 (25.1)	1,517 (8.4)	10 (0.1)		3,873 (21.4)	
16–17	11,898 (30.3)	7,421 (62.4)	2,692 (22.6)	1,721 (14.5)	64 (0.5)		2,603 (21.9)	
**Sex**								
Male^††^	18,907 (48.2)	13,658 (72.2)	3,713 (19.6)	1,505 (8.0)	31 (0.2)	0.07	4,369 (23.1)	0.03
Female	20,310 (51.7)	14,426 (71.0)	4,108 (20.2)	1,733 (8.5)	43 (0.2)		4,883 (24.0)	
**Race/Ethnicity**								
Hispanic	9,316 (23.7)	7,069 (75.9)	1,662 (17.8)	571 (6.1)	14 (0.2)	0.36	2,458 (26.4)	0.29
White, non-Hispanic	20,177 (51.4)	13,934 (69.1)	4,295 (21.3)	1,913 (9.5)	35 (0.2)		3,888 (19.3)	
Black, non-Hispanic	4,106 (10.4)	3,405 (82.9)	503 (12.3)	195 (4.7)	3 (0.1)		1,504 (36.6)	
Other, non-Hispanic^§§^	2,987 (7.6)	1,876 (62.8)	779 (26.1)	318 (10.6)	14 (0.5)		718 (24.0)	
Unknown	2,631 (6.7)	1,800 (68.4)	582 (22.1)	241 (9.2)	8 (0.3)		684 (26.0)	
**Chronic respiratory condition** ^¶¶^								
Yes^††^	3,183 (8.1)	2,160 (67.9)	728 (22.9)	284 (8.9)	11 (0.3)	0.11	456 (14.3)	0.17
No	36,034 (91.8)	25,924 (71.9)	7,093 (19.7)	2,954 (8.2)	63 (0.2)		8,796 (24.4)	
Chronic nonrespiratory condition***								
Yes^††^	1,815 (4.6)	1,260 (69.4)	372 (20.5)	178 (9.8)	5 (0.3)	0.05	379 (20.9)	0.03
No	37,402 (95.3)	26,824 (71.7)	7,449 (19.9)	3,060 (8.2)	69 (0.2)		8,873 (23.7)	

**Abbreviations:** ED = emergency department; ICD-9 = *International Classification of Diseases, Ninth Revision;* ICD-10 = *International Classification of Diseases, Tenth Revision;* SMD = standardized mean or proportion difference; UC = urgent care.

* Medical events with an encounter or discharge code consistent with COVID-19–like illness were included, using ICD-9 and ICD-10. Four categories of codes were considered: 1) acute respiratory illness, including COVID-19, respiratory failure, viral or bacterial pneumonia, asthma exacerbation, influenza, and viral illness not otherwise specified; 2) nonrespiratory COVID-19–like illness diagnoses including cause-unspecified gastroenteritis, thrombosis, and acute myocarditis; 3) respiratory signs and symptoms consistent with COVID-19–like illness, including hemoptysis, cough, dyspnea, painful respiration, or hypoxemia; and 4) signs and symptoms of acute febrile illness. One code in any of the four categories was sufficient for inclusion. Clinician-ordered molecular assays (e.g., real-time reverse transcription–polymerase chain reaction) for SARS-CoV-2 occurring ≤14 days before to <72 hours after the encounter date were included.

^†^ Vaccination was defined as having received the listed number of doses of COVID-19 Pfizer-BioNTech BNT162b2 vaccine ≥14 days (for 2 doses) or ≥7 days (for 3 doses) before the medical event index date, which was the date of respiratory specimen collection associated with the most recent positive or negative SARS-CoV-2 test result before medical event or the admission date if testing only occurred after the admission.

^§^ Partners contributing data on medical events were in California (vaccine availability: April 30, 2021), Colorado (May 22, 2021), Indiana (April 27, 2021), Minnesota and Wisconsin (April 21, 2021), New York (April 27, 2021), Oregon and Washington (April 28, 2021), Texas (March 29, 2021), Utah (April 9, 2021). The study period began in September 2021 for partners located in Texas. For adolescents aged 16–17 years, the study period began when COVID-19 vaccines became available to all persons aged ≥16 years at each study site. For children aged 5–11 and persons aged 12–15 years, the study period began 5 weeks after the Pfizer-BioNTech vaccine was authorized for each age group (November 2, 2021, and May 12, 2021, respectively).

^¶^ An absolute SMD ≥0.20 indicates a nonnegligible difference in variable distributions between medical events for vaccinated versus unvaccinated patients; single SMD calculated by averaging pairwise comparisons of each vaccinated category versus unvaccinated and separately for patients with SARS-CoV-2–positive versus SARS-CoV-2–negative test results. For example, the age SMD calculation comparing unvaccinated versus different vaccinated categories was generated by averaging the pairwise SMD calculations for unvaccinated and 2 doses (14–149 days earlier), unvaccinated and 2 doses (≥150 days earlier), and unvaccinated and 3 doses (≥7 days earlier). In addition, the age SMD calculation comparing negative SARS-CoV-2 test result and positive SARS-CoV-2 test result was generated by directly calculating the SMD for negative SARS-CoV-2 test result and positive SARS-CoV-2 test result.

** Estimated date of Delta and Omicron predominance at contributing sites: California (Delta: June 23, 2021; Omicron: December 21, 2021); Colorado (Delta: June 3, 2021; Omicron: December 19, 2021); Indiana (Delta: June 23, 2021; Omicron: December 26, 2021); Minnesota and Wisconsin (Delta: June 28, 2021; Omicron: December 25, 2021); New York (Delta: June 30, 2021; Omicron: December 18, 2021); Oregon and Washington (Delta: June 30, 2021; Omicron: December 24, 2021); Texas (Delta: July 3, 2021; Omicron: December 16, 2021); Utah (Delta: June 1, 2021; Omicron December 24, 2021). Pre-Delta refers to the period before Delta predominance.

^††^ Indicates the reference group used for standardized mean or proportion difference calculations for dichotomous variables.

^§§^ Other race includes Asian, Native Hawaiian or other Pacific islander, American Indian or Alaska Native, Other not listed, and multiple races.

^¶¶^ Chronic respiratory condition was defined as the presence of discharge code for asthma, sleep apnea, or other lung disease using ICD-9 and ICD-10 diagnosis codes.

*** Chronic nonrespiratory condition was defined as the presence of discharge code for heart failure, ischemic heart disease, hypertension, other heart disease, stroke, other cerebrovascular disease, diabetes type I or II, other diabetes, metabolic disease, clinical obesity, clinically underweight, renal disease, liver disease, blood disorder, immunosuppression, organ transplant, cancer, neurologic disorder, musculoskeletal disorder, Down Syndrome, congenital heart disease, neurologic conditions, muscular dystrophy, sickle cell disease, prematurity (<24 weeks), developmental delay, technology dependence, or chronic gastrointestinal disease/irritable bowel syndrome.

Among children aged 5–11 years, VE of 2 doses received 14–67 days earlier against COVID-19–associated ED and UC encounters was 46% (Table 2). Among adolescents aged 12–15 and 16–17 years, VE of 2 doses 14–149 days earlier against COVID-19–associated ED and UC encounters was 83% and 76%, respectively; VE was significantly lower for 2 doses received ≥150 days earlier (38% and 46%, respectively). Among adolescents aged 16–17 years, VE after receipt of a third dose ≥7 days earlier increased to 86%, significantly higher than the VE of 2 doses received ≥150 days earlier. The number of observations was insufficient to estimate 3-dose VE for adolescents aged 12–15 years. Compared with the Delta predominant period, estimated 2-dose VE for adolescents aged 12–15 and 16–17 years declined significantly once Omicron became the predominant variant: among adolescents aged 16–17 years, VE of 2 doses received ≥150 days earlier against COVID-19–associated ED and UC encounters declined from 77% during Delta predominance to a null VE (–3%) during Omicron predominance; however, effectiveness of a third dose received ≥7 days earlier against COVID-19–associated ED and UC encounters during Omicron predominance was 81%. Among children aged 5–11 years, VE of 2 doses received 14–67 days earlier against COVID-19–associated ED and UC encounters during Omicron predominance was 51%.

**TABLE 2:** COVID-19 Pfizer-BioNTech vaccine effectiveness* against laboratory-confirmed COVID-19–associated^†^ emergency department and urgent care clinic encounters and hospitalizations among children aged 5–17 years, by number and timing of vaccine doses^§^ and predominant circulating SARS-CoV-2 variant — VISION Network, 10 states,^¶^ April 2021 to January 2022

Encounter type/Vaccination status	Total	SARS-CoV-2 test-positive, no. (%)	VE %* (95% CI)
**ED or UC encounters during Delta or Omicron predominance, by age group**			
**5–11 yrs**			
Unvaccinated (Ref)	8,599	2,652 (30.8)	—
2 doses (14–67 days earlier)	582	124 (21.3)	46 (24–61)
**12–15 yrs**			
Unvaccinated (Ref)	12,064	3,238 (26.8)	—
2 doses (14–149 days earlier)	4,547	254 (5.6)	83 (80–85)
2 doses (≥150 days earlier)	1,517	378 (24.9)	38 (28–48)
3 doses (≥7 days earlier)	10	3 (30)	NC
**16–17 yrs**			
Unvaccinated (Ref)	7,421	2,068 (27.9)	—
2 doses (14–149 days earlier)	2,692	193 (7.2)	76 (71–80)
2 doses (≥150 days earlier)	1,721	329 (19.1)	46 (36–54)
3 doses (≥7 days earlier)	64	13 (20.3)	86 (73–93)
**ED or UC encounters, by age group and predominant variant**			
5–11 yrs**			
**Omicron predominant** ^††^			
Unvaccinated (Ref)	5,938	2,409 (40.6)	—
2 doses (14–67 days earlier)	486	118 (24.3)	51 (30–65)
**12–15 yrs**			
**Delta predominant** ^††^			
Unvaccinated (Ref)	9,633	1,978 (20.5)	—
2 doses (14–149 days earlier)	4,060	80 (2.0)	92 (89–94)
2 doses (≥150 days earlier)	798	32 (4.0)	79 (68–86)
**Omicron predominant** ^††^			
Unvaccinated (Ref)	2,336	1,254 (53.7)	—
2 doses (14–149 days earlier)	472	174 (36.9)	45 (30–57)
2 doses (≥150 days earlier)	719	346 (48.1)	−2 (−25–17)
3 doses (≥7 days earlier)	10	3 (30.0)	NC
**16–17 yrs**			
**Delta predominant** ^††^			
Unvaccinated (Ref)	5,302	1,191 (22.5)	—
2 doses (14–149 days earlier)	2,340	78 (3.3)	85 (81–89)
2 doses (≥150 days earlier)	1,156	47 (4.1)	77 (67–84)
3 doses (≥7 days earlier)	2	0 (—)	NC
**Omicron predominant** ^††^			
Unvaccinated (Ref)	1,363	771 (56.6)	—
2 doses (14–149 days earlier)	263	114 (43.4)	34 (8–53)
2 doses (≥150 days earlier)	565	282 (49.9)	−3 (−30–18)
3 doses (≥7 days earlier)	62	13 (21.0)	81 (59–91)
**Hospitalizations during Delta or Omicron predominance, by age group**			
**5–11 yrs**			
Unvaccinated (Ref)	262	59 (22.5)	—
2 doses (14–67 days earlier)	23	2 (8.7)	74 (−35–95)
**12–15 yrs**			
Unvaccinated (Ref)	496	149 (30)	—
2 doses (14–149 days earlier)	182	7 (3.8)	92 (79–97)
2 doses (≥150 days earlier)	63	13 (20.6)	73 (43–88)
**16–17 yrs**			
Unvaccinated (Ref)	437	136 (31.1)	—
2 doses (14–149 days earlier)	150	7 (4.7)	94 (87–97)
2 doses (≥150 days earlier)	82	14 (17.1)	88 (72–95)
3 doses (≥7 days earlier)	4	1 (25.0)	NC

**Abbreviations:** ED = emergency department; NC = not calculated; Ref = referent group; UC = urgent care; VE = vaccine effectiveness.

* VE was calculated as [1 − odds ratio] x 100%, estimated using a test-negative design, adjusted for age, geographic region, calendar time (days since January 1, 2021), and local virus circulation (percentage of SARS-CoV-2–positive results from testing within the counties surrounding the facility on the date of the encounter) and weighted for inverse propensity to be vaccinated or unvaccinated. Generalized boosted regression trees were used to estimate the propensity to be vaccinated based on sociodemographic characteristics, underlying medical conditions, and facility characteristics.

^†^ Medical events with an encounter or discharge code consistent with COVID-19–like illness were included, using *International Classification of Disease, Ninth Revision* and *International Classification of Diseases, Tenth Revision*. Four categories of codes were considered: 1) acute respiratory illness, including COVID-19, respiratory failure, viral or bacterial pneumonia, asthma exacerbation, influenza, and viral illness not otherwise specified; 2) nonrespiratory COVID-19–like illness diagnoses including cause-unspecified gastroenteritis, thrombosis, and acute myocarditis; 3) respiratory signs and symptoms consistent with COVID-19–like illness, including hemoptysis, cough, dyspnea, painful respiration, or hypoxemia; and 4) signs and symptoms of acute febrile illness. One code in any of the four categories was sufficient for inclusion. Clinician-ordered molecular assays (e.g., real-time reverse transcription–polymerase chain reaction) for SARS-CoV-2 occurring ≤14 days before to <72 hours after the encounter date were included.

^§^ Vaccination was defined as having received the listed number of doses of an mRNA-based COVID-19 Pfizer-BioNTech vaccine ≥14 days (for 2 doses) or ≥7 days (for 3 doses) before the medical event index date, which was the date of respiratory specimen collection associated with the most recent positive or negative SARS-CoV-2 test result before medical event or the admission date if testing only occurred after the admission.

^¶^ Partners contributing data on medical events were in California (vaccine availability: April 30, 2021), Colorado (May 22, 2021), Indiana (April 27, 2021), Minnesota and Wisconsin (April 21, 2021), New York (April 27, 2021), Oregon and Washington (April 28, 2021), Texas (March 29, 2021), Utah (April 9, 2021). The study period began in September 2021 for partners located in Texas. For adolescents aged 16–17 years, the study period began when COVID-19 vaccines became available to all those aged ≥16 years at each study site. For children aged 5–11 and persons aged 12–15 years, the study period began 5 weeks after the Pfizer-BioNTech vaccine was authorized for each age group (November 2, 2021, and May 12, 2021, respectively).

** VE during the period of Delta predominance was not calculated for children aged 5–11 years because of the short eligibility interval in this age group during that time.

^††^ Estimated date of Delta and Omicron predominance at contributing sites: California (Delta: June 23, 2021; Omicron: December 21, 2021); Colorado (Delta: June 3, 2021; Omicron: December 19, 2021); Indiana (Delta: June 23, 2021; Omicron: December 26, 2021); Minnesota and Wisconsin (Delta: June 28, 2021; Omicron: December 25, 2021); New York (Delta: June 30, 2021; Omicron: December 18, 2021); Oregon and Washington (Delta: June 30, 2021; Omicron: December 24, 2021); Texas (Delta: July 3, 2021; Omicron: December 16, 2021); Utah (Delta: June 1, 2021; Omicron December 24, 2021).

## Hospitalizations

Among 1,699 eligible hospitalizations at 164 hospitals, 16.8%, 43.6%, and 39.6% were among children and adolescents aged 5–11, 12–15 and 16–17 years, respectively (Table 3). Most hospitalizations of adolescents aged 12–15 years (613 [82.7%]) and 16–17 years (476 [70.7%]) occurred during Delta predominance, whereas two thirds of hospitalizations among children aged 5–11 years (190 [66.7%]) occurred during Omicron predominance.

**TABLE 3:** Characteristics of hospitalizations among children aged 5–17 years with COVID-19–like illness* by COVID-19 Pfizer-BioNTech vaccination status^†^ and SARS-CoV-2 test result — 10 states,^§^ April 2021 to January 2022

Characteristic	Total, no. (column %)	No. (row %)				SMD^¶^	No. (row %)	SMD^¶^
		Pfizer-BioNTech vaccination status					Positive SARS-CoV-2 test result	
		Unvaccinated	2 doses (14–149 days earlier)	2 doses (≥150 days earlier)	3 doses (≥7 days earlier)			
**All hospitalizations**	**1,699**	**1,195 (70.3)**	**355 (20.9)**	**145 (8.5)**	**4 (0.2)**	**–**	**388 (22.8)**	**–**
Variant predominance period**								
Pre-Delta	110 (6.4)	91 (82.7)	19 (17.3)	0 (—)	0 (—)	1.11	13 (11.8)	0.46
B.1.617.2 (Delta)	1,184 (69.6)	812 (68.6)	288 (24.3)	84 (7.1)	0 (—)		224 (18.9)	
B.1.1.529 (Omicron)	405 (23.8)	292 (72.1)	48 (11.9)	61 (15.1)	4 (1.0)		151 (37.3)	
**Site**								
Baylor Scott & White Health	189 (11.1)	167 (88.4)	14 (7.4)	7 (3.7)	1 (0.5)	1.4	42 (22.2)	0.49
Columbia University	162 (9.5)	118 (72.8)	35 (21.6)	9 (5.6)	0 (—)		19 (11.7)	
HealthPartners	40 (2.3)	22 (55.0)	13 (32.5)	5 (12.5)	0 (—)		3 (7.5)	
Intermountain Healthcare	403 (23.7)	261 (64.8)	97 (24.1)	45 (11.2)	0 (—)		113 (28.0)	
Kaiser Permanente Northern California	265 (15.5)	115 (43.4)	105 (39.6)	42 (15.8)	3 (1.1)		31 (11.7)	
Kaiser Permanente Northwest	57 (3.3)	32 (56.1)	21 (36.8)	4 (7.0)	0 (—)		9 (15.8)	
Regenstrief Institute	371 (21.8)	315 (84.9)	37 (10.0)	19 (5.1)	0 (—)		121 (32.6)	
University of Colorado	212 (12.4)	165 (77.8)	33 (15.6)	14 (6.6)	0 (—)		50 (23.6)	
**Age group, yrs**								
5–11	285 (16.8)	262 (91.9)	23 (8.1)	0 (—)	0 (—)	1.03	61 (21.4)	0.04
12–15	741 (43.6)	496 (66.9)	182 (24.6)	63 (8.5)	0 (—)		169 (22.8)	
16–17	673 (39.6)	437 (64.9)	150 (22.3)	82 (12.2)	4 (0.6)		158 (23.5)	
**Sex**								
Male^††^	805 (47.3)	570 (70.8)	171 (21.2)	61 (7.6)	3 (0.4)	0.24	161 (20.0)	0.15
Female	894 (52.6)	625 (69.9)	184 (20.6)	84 (9.4)	1 (0.1)		227 (25.4)	
**Race/Ethnicity**								
Hispanic	454 (26.7)	326 (71.8)	100 (22.0)	28 (6.2)	0 (—)	0.53	93 (20.5)	0.13
White, non-Hispanic	733 (43.1)	493 (67.3)	159 (21.7)	79 (10.8)	2 (0.3)		186 (25.4)	
Black, non-Hispanic	242 (14.2)	193 (79.8)	31 (12.8)	17 (7.0)	1 (0.4)		50 (20.7)	
Other, non-Hispanic^§§^	207 (12.1)	131 (63.3)	58 (28.0)	17 (8.2)	1 (0.5)		45 (21.7)	
Unknown	63 (3.7)	52 (82.5)	7 (11.1)	4 (6.3)	0 (—)		14 (22.2)	
**Chronic respiratory condition^¶¶^**								
Yes^††^	1,090 (64.1)	756 (69.4)	249 (22.8)	82 (7.5)	3 (0.3)	0.18	150 (13.8)	0.70
No	609 (35.8)	439 (72.1)	106 (17.4)	63 (10.3)	1 (0.2)		238 (39.1)	
Chronic nonrespiratory condition***								
Yes^††^	930 (54.7)	647 (69.6)	202 (21.7)	78 (8.4)	3 (0.3)	0.17	207 (22.3)	0.04
No	769 (45.2)	548 (71.3)	153 (19.9)	67 (8.7)	1 (0.1)		181 (23.5)	

**Abbreviations:** ICD-9 = *International Classification of Diseases, Ninth Revision;* ICD-10 = *International Classification of Diseases, Tenth Revision*; SMD = standardized mean or proportion difference.

* Medical events with an encounter or discharge code consistent with COVID-19–like illness were included, using ICD-9 and ICD-10. Four categories of codes were considered: 1) acute respiratory illness, including COVID-19, respiratory failure, viral or bacterial pneumonia, asthma exacerbation, influenza, and viral illness not otherwise specified; 2) nonrespiratory COVID-19–like illness diagnoses including cause-unspecified gastroenteritis, thrombosis, and acute myocarditis; 3) respiratory signs and symptoms consistent with COVID-19–like illness, including hemoptysis, cough, dyspnea, painful respiration, or hypoxemia; and 4) signs and symptoms of acute febrile illness. One code in any of the four categories was sufficient for inclusion. Clinician-ordered molecular assays (e.g., real-time reverse transcription–polymerase chain reaction) for SARS-CoV-2 occurring ≤14 days before to <72 hours after the encounter date were included.

^†^ Vaccination was defined as having received the listed number of doses of COVID-19 Pfizer-BioNTech vaccine ≥14 days (for 2 doses) or ≥7 days (for 3 doses) before the medical event index date, which was the date of respiratory specimen collection associated with the most recent positive or negative SARS-CoV-2 test result before medical event or the admission date if testing only occurred after the admission.

^§^ Partners contributing data on medical events were in California (vaccine availability: April 30, 2021), Colorado (May 22, 2021), Indiana (April 27, 2021), Minnesota and Wisconsin (April 21, 2021), New York (April 27, 2021), Oregon and Washington (April 28, 2021), Texas (March 29, 2021), Utah (April 9, 2021). The study period began in September 2021 for partners located in Texas. For adolescents aged 16–17 years, the study period began when COVID-19 vaccines became available to all those aged ≥16 years at each study site. For children aged 5–11 and persons aged 12–15 years, the study period began 5 weeks after the Pfizer-BioNTech vaccine was authorized for each age group (November 2, 2021, and May 12, 2021, respectively).

^¶^ An absolute SMD ≥0.20 indicates a nonnegligible difference in variable distributions between medical events for vaccinated versus unvaccinated patients; single SMD calculated by averaging pair-wise comparisons of each vaccinated category versus unvaccinated and separately for patients with SARS-CoV-2–positive versus SARS-CoV-2–negative test results. For example, the age SMD calculation comparing unvaccinated versus different vaccinated categories was generated by averaging the pairwise SMD calculations for unvaccinated and 2 doses (14–149 days earlier), unvaccinated and 2 doses (≥150 days earlier), and unvaccinated and 3 doses (≥7 days). In addition, the age SMD calculation comparing negative SARS-CoV-2 test result and positive SARS-CoV-2 test result was generated by directly calculating the SMD for negative SARS-CoV-2 test result and positive SARS-CoV-2 test result.

** Estimated date of Delta and Omicron predominance at contributing sites: California (Delta: June 23, 2021; Omicron: December 21, 2021); Colorado (Delta: June 3, 2021; Omicron: December 19, 2021); Indiana (Delta: June 23, 2021; Omicron: December 26, 2021); Minnesota and Wisconsin (Delta: June 28, 2021; Omicron: December 25, 2021); New York (Delta: June 30, 2021; Omicron: December 18, 2021); Oregon and Washington (Delta: June 30, 2021; Omicron: December 24, 2021); Texas (Delta: July 3, 2021; Omicron: December 16, 2021); Utah (Delta: June 1, 2021; Omicron December 24, 2021). Pre-Delta refers to the period before Delta predominance.

^††^ Indicates the reference group used for SMD calculations for dichotomous variables.

^§§^ Other race includes Asian, Native Hawaiian or other Pacific islander, American Indian or Alaska Native, Other not listed, and multiple races.

^¶¶^ Chronic respiratory condition was defined as the presence of discharge code for asthma, sleep apnea, or other lung disease using ICD-9 and ICD-10 diagnosis codes.

*** Chronic nonrespiratory condition was defined as the presence of discharge code for heart failure, ischemic heart disease, hypertension, other heart disease, stroke, other cerebrovascular disease, diabetes type I or II, other diabetes, metabolic disease, clinical obesity, clinically underweight, renal disease, liver disease, blood disorder, immunosuppression, organ transplant, cancer, neurologic disorder, musculoskeletal disorder, Down Syndrome, congenital heart disease, neurologic conditions, muscular dystrophy, sickle cell disease, prematurity (<24 weeks), developmental delay, technology dependence, or chronic gastrointestinal disease/irritable bowel syndrome.

Among children aged 5–11 years, estimated VE of 2 vaccine doses received 14–67 days earlier against COVID-19–associated hospitalization was 74%, with wide confidence intervals that included zero (95% CI = –35% to 95%) (Table 2). Among adolescents aged 12–15 and 16–17 years, VE of 2 doses received 14–149 days earlier was 92% and 94%, respectively, and VE of 2 doses received ≥150 days earlier was 73% and 88%, respectively. Differences by time since vaccination were not statistically significant.

## Discussion

In a multistate analysis of 39,217 ED and UC encounters with COVID-19–like illness among nonimmunocompromised patients aged 5–17 years through January 29, 2022, estimates of Pfizer-BioNTech VE against COVID-19–associated ED and UC encounters varied by time since vaccination and by predominant circulating SARS-CoV-2 variant. Among adolescents aged 12–17 years during the full study period including pre-Delta, Delta, and Omicron predominant periods, 2-dose VE estimates were higher (76%–83%) 14–149 days after receipt of a second dose, and significantly lower (38%–46%) at ≥150 days postvaccination. However, a third vaccine dose restored VE against COVID-19–associated ED or UC encounters to 86% among adolescents aged 16–17 years. Among children aged 5–11 years during the full study period, VE of 2 doses (14–67 days earlier) against COVID-19–associated ED or UC encounters was 46%, which was significantly lower than overall estimates for adolescents aged 12–17 years. However, most encounters among children aged 5–11 years occurred during Omicron predominance, when VE significantly declined for adolescents aged 12–17 years. During Omicron predominance, VE of a second dose received 14–149 days earlier was 45% and 34% for adolescents aged 12–15 and 16–17 years, respectively, suggesting that the lower VE observed among children aged 5–11 years was likely driven by the predominant variant rather than differences in VE across age groups. During Omicron predominance, there was no evidence of protection for adolescents aged 12–17 years from 2 doses received ≥150 days earlier; however, a third vaccine restored VE to 81% among adolescents aged 16–17 years. 

Receipt of 2 Pfizer-BioNTech vaccine doses in persons aged 12–17 years provided a high level of protection (>90%) against COVID-19–associated hospitalizations within 149 days of receipt of the second dose. VE point estimates for second dose received ≥150 days earlier were 73% to 88%; however, differences by time since vaccination were not statistically significant. Additional data are needed to better understand duration of protection against COVID-19–associated hospitalization in adolescents aged 12–17 years, the protection from 3 doses, and the level of protection among children aged 5–11 years.

These findings are consistent with previously published data showing high effectiveness of the Pfizer-BioNTech vaccine among adolescents before Omicron became the predominant variant (*4*–*6*), and with data from adults demonstrating relatively higher protection against more severe outcomes (*7*). These findings are also consistent with data showing a decline in mRNA VE over time since receipt of the second dose among adolescents and adults (*8*–*10*). The findings in this report also align with studies among adults that report lower VEs during Omicron variant predominance (*9*,*10*) and an increase in VE after receipt of a third vaccine dose (*9*,*10*).

The findings in this report are subject to at least six limitations. First, comparison of VE estimates between age groups should be made with caution because of differences in the timing of vaccine availability and predominant variants when the vaccine became available to different age groups. Second, statistical power for estimating VE against COVID-19–associated hospitalizations was limited, resulting in wide CIs for some groups, particularly children aged 5–11 years. Third, among adolescents aged 16–17, the estimated 3-dose VE was based on a relatively short period after vaccination. Fourth, despite adjustments to balance the differences between unvaccinated and vaccinated persons, unmeasured and residual confounding (e.g., mask use and physical distancing) might have biased the estimates. Fifth, genetic characterization of patients’ viruses was not available, and Delta and Omicron predominance periods were based on surveillance data. Finally, although the facilities in this study serve heterogeneous populations in 10 states, the findings might not be generalizable to the U.S. population.

This report provides real-world evidence of protection by the Pfizer-BioNTech vaccine against COVID-19–associated ED and UC encounters and hospitalizations among children and adolescents aged 5–17 years and supports the role of third (booster) doses in maintaining high levels of VE in the setting of Omicron predominance. All eligible children and adolescents should remain up to date with recommended COVID-19 vaccinations, including a booster dose for those aged 12–17 years.^§§§^

**Summary**

**What is already known about this topic?**

Two doses of Pfizer-BioNTech vaccine provided protection against COVID-19 in persons aged 12–17 years during Delta predominance, but data during Omicron predominance and among children aged 5–11 years are lacking.

**What is added by this report?**

Two doses protect against COVID-19–associated emergency department and urgent care encounters among children and adolescents. However, vaccine effectiveness (VE) was lower during Omicron predominance and decreased with time since vaccination; a booster dose restored VE to 81% among adolescents aged 16–17 years. Overall, 2-dose VE against COVID-19–associated hospitalization was 73%–94%.

**What are the implications for public health practice?**

All eligible children and adolescents should remain up to date with recommended COVID-19 vaccinations, including a booster dose for those aged 12–17 years.

## Footnotes

* Funded by CDC, the VISION Network includes Baylor Scott & White Health (Texas), Columbia University Irving Medical Center (New York), HealthPartners (Minnesota and Wisconsin), Intermountain Healthcare (Utah), Kaiser Permanente Northern California (California), Kaiser Permanente Northwest (Oregon and Washington), Regenstrief Institute (Indiana), and University of Colorado (Colorado).

† The data in these analyses come from 306 ED and UC clinics and 164 hospitals.

§ The study period at Baylor Scott and White Health began on September 11, 2021.

¶ Medical events with an encounter or discharge code consistent with COVID-19–like illness were included, using *International Classification of Disease, Ninth Revision* and *International Classification of Diseases, Tenth Revision* (ICD-10). Four categories of codes were considered: 1) acute respiratory illness, including COVID-19, respiratory failure, viral or bacterial pneumonia, asthma exacerbation, influenza, and viral illness not otherwise specified; 2) nonrespiratory COVID-19–like illness diagnoses including cause-unspecified gastroenteritis, thrombosis, and acute myocarditis; 3) respiratory signs and symptoms consistent with COVID-19–like illness, including hemoptysis, cough, dyspnea, painful respiration, or hypoxemia; 4) signs and symptoms of acute febrile illness. One code in any of the four categories was sufficient for inclusion. Clinician-ordered molecular assays (e.g., real-time reverse transcription–polymerase chain reaction) for SARS-CoV-2 occurring ≤14 days before to <72 hours after the encounter date were included.

** The Food and Drug Administration (FDA) issued an Emergency Use Authorization (EUA) for the Pfizer-BioNTech vaccine for persons aged ≥16 years on December 11, 2020 (https://www.fda.gov/news-events/press-announcements/fda-takes-key-action-fight-against-covid-19-issuing-emergency-use-authorization-first-covid-19), and CDC recommended the Pfizer-BioNTech vaccine on December 12, 2020 (https://www.cdc.gov/media/releases/2020/s1213-covid-vaccine.html). CDC recommended a booster dose for adolescents aged 16–17 years on December 9, 2021(https://www.cdc.gov/media/releases/2021/s1208-16-17-booster.html).

†† FDA amended the EUA for the Pfizer-BioNTech vaccine to include adolescents aged 12–15 years on May 10, 2021 (https://www.fda.gov/news-events/press-announcements/coronavirus-covid-19-update-fda-authorizes-pfizer-biontech-covid-19-vaccine-emergency-use), and CDC recommended the Pfizer-BioNTech vaccine in this age group on May 12, 2021 (https://www.cdc.gov/media/releases/2021/s0512-advisory-committee-signing.html). FDA authorized the EUA for the Pfizer-BioNTech vaccine for children aged 5–11 years on October 29, 2021 (https://www.fda.gov/news-events/press-announcements/fda-authorizes-pfizer-biontech-covid-19-vaccine-emergency-use-children-5-through-11-years-age), and CDC recommended the Pfizer-BioNTech vaccine for this age group on November 2, 2021 (https://www.cdc.gov/media/releases/2021/s1102-PediatricCOVID-19Vaccine.html). On January 5, 2022, CDC expanded its recommendation for a booster 5 months after receipt of the second dose of the Pfizer-BioNTech vaccine to include adolescents aged 12–15 years (https://www.cdc.gov/media/releases/2022/s0105-Booster-Shot.html).

§§ Estimated date of Delta and Omicron predominance at contributing sites: California (Delta: June 23, 2021; Omicron: December 21, 2021); Colorado (Delta: June 3, 2021; Omicron: December 19, 2021); Indiana (Delta: June 23, 2021; Omicron: December 26, 2021); Minnesota and Wisconsin (Delta: June 28, 2021; Omicron: December 25, 2021); New York (Delta: June 30, 2021; Omicron: December 18, 2021); Oregon and Washington (Delta: June 30, 2021; Omicron: December 24, 2021); Texas (Delta: July 3, 2021; Omicron: December 16, 2021); Utah (Delta: June 1, 2021; Omicron December 24, 2021). Pre-Delta refers to the period before Delta predominance.

¶¶ Immunocompromised status was defined using ICD-9 and ICD-10 as the presence of discharge codes for solid malignancy, hematologic malignancy, rheumatologic or inflammatory disorder, other intrinsic immune condition or immunodeficiency, or organ or stem cell transplant.

*** With a test-negative design, vaccine performance is assessed by comparing the odds of antecedent vaccination among case-patients with acute laboratory-confirmed COVID-19 and control-patients without acute COVID-19. This odds ratio was adjusted for age, geographic region, calendar time (days from January 1), and local virus circulation in the community and weighted for inverse propensity to be vaccinated or unvaccinated.

††† 45 C.F.R. part 46; 21 C.F.R. part 56.

§§§ https://www.cdc.gov/coronavirus/2019-ncov/vaccines/recommendations/children-teens.html (Accessed January 11, 2022).
